# Machine learning‐based identification and related features of depression in patients with diabetes mellitus based on the Korea National Health and Nutrition Examination Survey: A cross-sectional study

**DOI:** 10.1371/journal.pone.0288648

**Published:** 2023-07-13

**Authors:** Ji-Yoon Lee, Doyeon Won, Kiheon Lee

**Affiliations:** 1 Department of Health Science and Technology, Graduate School of Convergence Science and Technology, Seoul National University, Seoul, Republic of Korea; 2 Department of Family Medicine, Seoul National University Bundang Hospital, Seongnam, Republic of Korea; 3 Department of Family Medicine, Seoul National University College of Medicine, Seoul, Republic of Korea; Hangil Eye Hospital / Catholic Kwandong University College of Medicine, REPUBLIC OF KOREA

## Abstract

Patients with diabetes mellitus (DM) are twice as likely as nondiabetic individuals to develop depression, which is a prevalent but often undiagnosed psychiatric comorbidity. Patients with DM who are depressed have poor glycemic control, worse quality of life, increased risk of diabetic complications, and higher mortality rate. The present study aimed to develop machine learning (ML) models that identify depression in patients with DM, determine the best performing model by evaluating multiple ML algorithms, and investigate features related to depression. We developed six ML models, including random forest, K-nearest neighbor, support vector machine (SVM), Adaptive Boosting, light gradient-boosting machine, and Extreme Gradient Boosting, based on the Korea National Health and Nutrition Examination Survey. The results showed that the SVM model performed well, with a cross-validated area under the receiver operating characteristic curve of 0.835 (95% confidence interval [CI] = 0.730–0.901). Thirteen features were related to depression in patients with DM. Permutation feature importance showed that the most important feature was subjective health status, followed by level of general stress awareness; stress recognition rate; average monthly income; triglyceride (mg/dL) level; activity restriction status; European quality of life (EuroQoL): usual activity and lying in a sickbed in the past 1 month; EuroQoL: pain / discomfort, self-care, and physical discomfort in the last 2 weeks; and EuroQoL: mobility and chewing problems. The current findings may offer clinicians a better understanding of the relationship between DM and depression using ML approaches and may be an initial step toward developing a more predictive model for the early detection of depressive symptoms in patients with DM.

## Introduction

Diabetes mellitus (DM) is one of the fastest growing health problems of the twenty-first century worldwide. According to the International Diabetes Federation, the global DM prevalence in individuals aged 20–79 years in 2021 was approximately 10.5% (536.6 million individuals), increasing to 12.2% (783.2 million individuals) in 2045 [1]. In South Korea, the estimated prevalence of Korean adults aged ≥ 30 years with DM was 16.7% in 2020 [2]. The per capita cost burden of patients with DM is two to four times greater than that of nondiabetic patients, and a significant amount of the expenses are attributable to managing comorbid illnesses [3].

Depression is a common psychiatric comorbidity in patients with DM. The prevalence of depressive disorders in DM generally ranges from 10% to 15%, which is approximately twice as high as the prevalence of depression in nondiabetic individuals [4]. Coronavirus disease 2019 (COVID-19) has profoundly affected all aspects of human life globally since 2020. The COVID-19 pandemic has affected the prevention and treatment of comorbid conditions, that is, depression and DM [5]. Depression is related to adverse effects in individuals with DM, including decreased glycemic control, nonadherence to therapy, poor metabolic control, and increased risk of vascular problems (e.g., diabetic retinopathy, neuropathy, and macrovascular complications). Additionally, depression in patients with diabetes often persists and is related to a higher risk of death, increased disability, lower quality of life, and somatic symptom burden. A previous study has demonstrated an increased risk of dementia for patients with depression [6]. Compared with patients with type 2 DM (T2DM) without depression at baseline, those with depression have higher longitudinal risk of clinically significant post-diabetic micro- and macrovascular problems [7]. Therefore, treating depression in patients with DM is crucial for managing DM and its complications.

Using machine learning (ML), data-driven methods can enhance the accuracy (ACC) of identification by utilizing a large dataset to impartially detect new related features. Several ML-based studies have been conducted to predict depression in patients with DM. Jin [8] developed a clinical forecasting model that predicts comorbid depression among patients using ML algorithms. They demonstrated that a logistic regression model with seven predictors, including female sex, Toobert diabetes self-care, total number of diabetes complications, previous diagnosis of major depressive disorder, number of International Statistical Classification of Diseases and Related Health Problems-9 diagnoses in the past 6 months, chronic pain, and self-rated health status, performed best among other models. Another study demonstrated that a model using a support vector machine (SVM) algorithm outperformed other models in predicting depression in patients with DM [9].

However, further studies using ML techniques for depression diagnosis, including various algorithms, study populations, and features, are required. DM and depression have various risk factors, and their associations have been revealed [10]. Therefore, the present study aimed to develop ML-based depression classifiers based on a national survey, the Korea National Health and Nutrition Examination Survey (KNHANES), and to clarify the important features to classify depression in patients with DM using ML approaches.

## Methods

### Datasets

The KNHANES, cross-sectional national monitoring system, has evaluated the health and nutritional condition of Koreans since 1998. It is a complex, stratified, and multistage probability cluster designed for the entire South Korean population [11]. The KNHANES is open-access data from the KNHANES from 2014 to 2020 were used to train the models. The data comprised 31,051 samples with 894 features from health questionnaires and examinations conducted from 2014 to 2020. The definition of DM was based on health examinations, including fasting blood glucose level ≥ 126 mg/dL, hemoglobin A1c (HbA1c) level ≥ 6.5%, self-reported medical history of DM, or medical treatment with oral antidiabetic medications or insulin. Among the 3,472 patients with DM, those diagnosed before the age of 30 years (n = 42) were excluded because of the possibility of type 1 DM (T1DM). The clinical and demographic characteristics of the dataset were analyzed using the chi-squared test and one-way analysis of variance. The significance level was set at *p* < 0.05.

### Ethics statement

All participants voluntarily participated and were provided written informed consent before conducting the survey. Researchers were shared with anonymized raw data. The present study was approved by the Institutional Review Board of Seoul National University Bundang Hospital (IRB No. X-2208-774-901).

### Primary outcome

**Fig 1:**
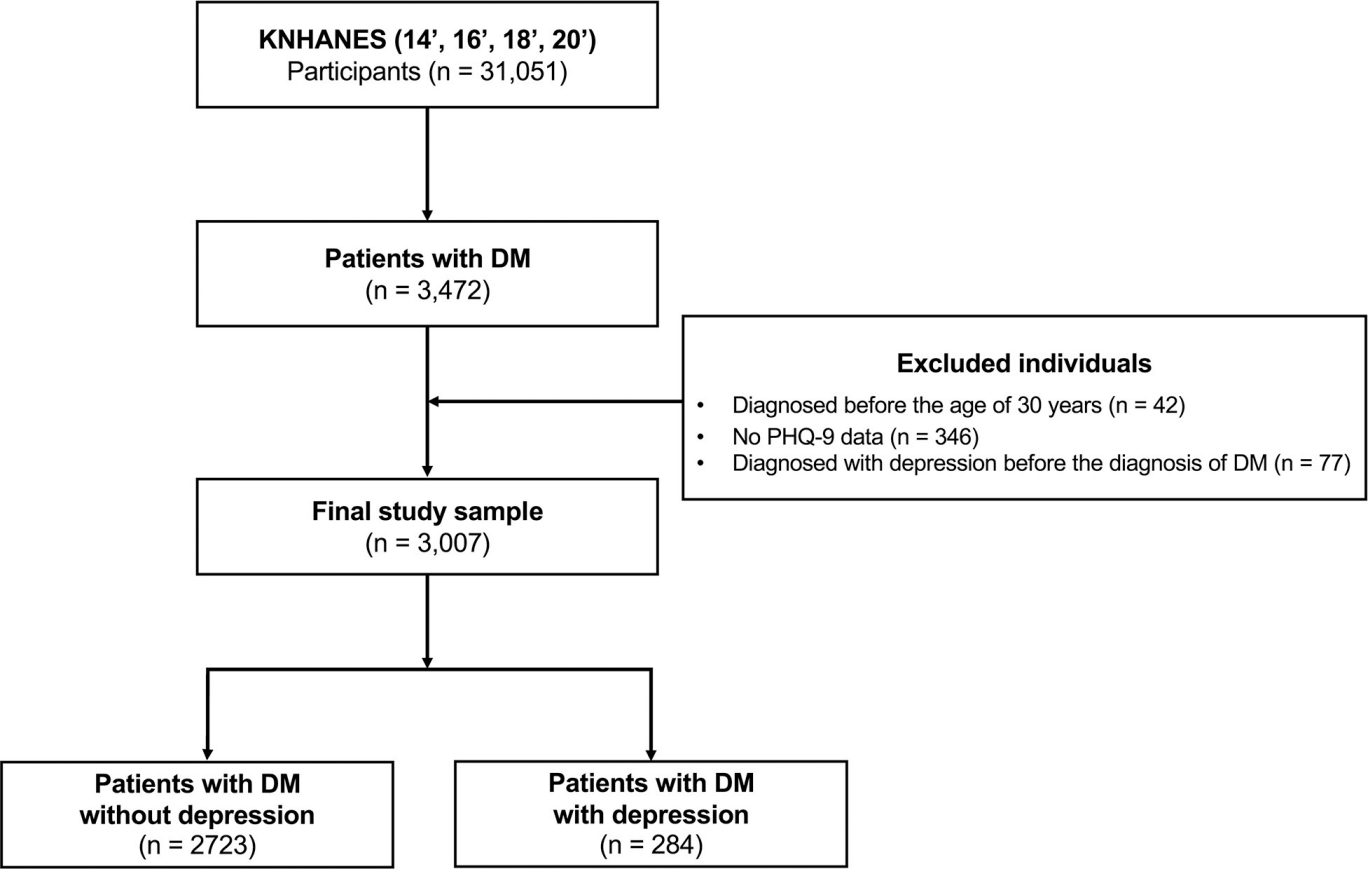
Flow diagram of sample selection. KNHANES, the Korea National Health and Nutrition Examination Survey; DM, diabetes mellitus; PHQ-9, Patient Health Questionnaire-9.

Depression was assessed using the standardized Korean version of Patient Health Questionnaire-9 (PHQ-9) [12]. The PHQ-9, which consists of nine questions and is based on the diagnostic criteria for depression in the Diagnostic and Statistical Manual of Mental Disorders IV, is a validated and reliable tool for identifying depression. In comparison to the Beck Depression Inventory-II [13], which is a reliable measure for assessing depression, the PHQ-9 has good internal consistency and responsiveness to change, and there is a moderate correlation between the two assessments [14]. In the present study, participants with a PHQ-9 score ≥ 10 were identified as having depression and had a self-reported medical history and treatment of depression [15,16]. As the PHQ-9 was only included in even years (14’, 16’, 18’, and 20’), we proceeded with the datasets. Those who completed the PHQ-9 were included in the final sample (n = 3,084). Patients diagnosed with depression before the diagnosis of DM (n = 77) were excluded. Finally, a total of 3,007 patients with DM (patients with DM without depression n = 2,723; patients with DM with depression n = 284) were included in the present study. The Fig 1 illustrated the flow chart outlining the procedure for selecting samples.

### Data processing and feature selection

Using the health questionnaires and health examinations of the KNHANES, first, we removed features that had ≥ 70% of missing features, and DM- and depression-related variables were excluded from the training and test sets. For example, the European quality of life (EuroQoL) questionnaire has five-dimension that assess self-reported problems (mobility, self-care, usual activities, pain/discomfort, and depression/anxiety) [17]. Therefore, the EuroQoL: anxiety/depression were excluded. Values of ‘8’ suggested ‘not applicable’ and ‘9’, ‘99’, ‘999’, and ‘9999’ suggested ‘I do not know about that question’ for variables regarded as missing values. We replaced the missing values with the mode for binary data and the median for numerical data. Numerical variables were standardized to avoid bias toward features with larger values and variances. Finally, 411 features were utilized for feature selection.

Subsequently, the Boruta algorithm was applied for feature selection. The Boruta algorithm, placed within the random forest (RF) classification method, offers an unbiased and consistent selection of important and unimportant information system properties [18]. By utilizing the Boruta method, we can identify features that exhibit significantly higher relevance with classification compared with randomly permuted features [19]. Thus, we selected features confirmed by the Boruta algorithm. Previous ML research using KNHANES demonstrated remarkable model performances by employing the Boruta algorithm for feature selection [20]. We used Python 3.8 (https://www.python.org/) and its compatible open-source packages.

### Model training

We trained the following six algorithms for depression classification: RF, K-nearest neighbor (KNN), linear SVM, light gradient-boosting machine (LightGBM), Extreme Gradient Boosting (XGBoost), and Adaptive Boosting (AdaBoost). First, we randomly divided the data into four training and one test sets using stratified cross-validation (CV) and applied the Synthetic Minority Oversampling TEchnique (SMOTE) to the training set to balance the ratio between depression and non-depression classes. Compared with other existing methods that rely on the random oversampling of instances, SMOTE addresses the overfitting problem by utilizing neighboring information to generate new artificial instances. By replicating and randomly increasing the minority class, SMOTE effectively balances the class distribution, as demonstrated in the studies by Chawla and Bowyer [21] and Azad and Bhushan [22]. Next, the algorithms were trained using four of the five subsets, and the classification performance of the model was measured on the remaining set. We trained the models with each algorithm using a grid search for hyperparameter tuning with five-fold CV. Finally, we iterated this procedure for each algorithm and calculated the average model performance indices, including the area under the receiver operating characteristic curve (AUC), ACC, recall, precision, and F1 score. Additionally, a 95% confidence interval (CI) for the AUC was calculated using bootstrapping methods.

### Feature importance

Feature importance was conducted with permutation importance, and the Python library ELI5 was used [23]. The topmost features were considered the most important, whereas those at the bottom were the least important. The first column of each row indicated the reduction in model ACC when random shuffling was applied, and the subsequent columns indicated the variation in ACC across multiple shufflings [24].

## Results

### Clinical and demographic characteristics

The clinical and demographic characteristics are presented in Table 1. Patients with DM with depression comprised 96 males and 188 females, and patients with DM without depression group comprised 1448 males and 1275 females. The chi-squared test was significantly important (*x*^2^ = 38.640, *p* < .001). Patients with DM with depression and patients with DM without depression were aged 64.110 ± 12.956 and 63.230 ± 11.731 years, respectively, and the difference was not statistically significant between the two groups (*F* = 1.442, *p* = 0.230). The average monthly income of patients with DM with depression was 192.084 ± 199.540 million won, which was significantly lower than that of patients with DM without depression (332.868 ± 298.230, *F* = 60.237, *p* < .001).

**Table 1:** The clinical and demographic characteristics of patients with diabetes mellitus with depression and without depression.

	Patients with DM with depression (n = 284)		Patients with DM without depression (n = 2723)		*x* ^*2*^	*p*
**Sex**						
male	96 (33.8%)		1448 (53.2%)		38.640***	< .001
female	188 (66.2%)		1275 (46.8%)			
**Whether diagnosed with DM by a physician**						
Yes	201 (70.8%)		1816 (66.7%)		1.942	0.163
No	83 (29.2%)		907 (33.3%)			
**Whether diagnosed with depression by a physician**						
Yes	103 (36.3%)		0 (0%)		1022.594***	< .001
No	181 (63.7%)		2723 (100%)			
**Whether received medical treatment of DM (Antidiabetics)**						
Yes	178 (97.3%)		1686 (98.6%)		1.935	0.164
No	5 (2.7%)		24 (1.4%)			
**Whether received medical treatment of DM (Insulin)**						
Yes	20 (10.9%)		128 (7.5%)		2.720	0.099
No	163 (89.1%)		1582 (92.5%)			
	**Mean**	**(S.D.)**	**Mean**	**(S.D.)**	***F***	***p***
**Age (year)**	64.110	(12.956)	63.230	(11.731)	1.442	0.230
**Average monthly income (million won)**	192.084	(199.540)	332.868	(298.230)	60.237***	< .001
**Age of DM diagnosis (year)**	54.551	(11.806)	56.332	(11.201)	4.468*	0.035
**Fasting Glucose (mg/dL)**	134.536	(37.991)	138.921	(40.370)	2.848	0.092
**HbA1c (%)**	6.934	(1.057)	7.147	(1.246)	7.025**	0.008
**PHQ-9 total score**	11.655	(6.093)	1.487	(1.942)	3733.031***	< .001

DM, diabetes mellitus; S.D, standard deviation; HbA1c, hemoglobin A1c; PHQ-9, Patient Health Questionnaire-9; *; *p* < 0.01

**; *p* < 0.001

***.p < 0.05

The mean age of diagnosis of patients with DM with depression (54.551 ± 11.806 years) was higher among patients with DM without depression (56.332 ± 11.201 years; *F* = 4.468, *p* = 0.035). HbA1c level was higher in patients with DM without depression (7.147 ± 1.249%) than in patients with DM without depression (6.934 ± 1.057%; *F* = 7.025, *p* = 0.008). In terms of PHQ-9 total score, patients with DM with depression scored 11.655 ± 6.093, whereas patients with DM without depression scored 1.487 ± 1.942 (*F* = 3733.031, *p* < .001).

### Selected features and feature characteristics

We selected 14 features confirmed by the Boruta algorithm. The selected variables were as follows: average monthly income, chewing problem, level of general stress awareness, subjective health status, physical discomfort in the last 2 weeks, height, triglyceride (mg/dL) level, EuroQoL (mobility, self-care, usual activities, pain/discomfort), lying in a sickbed in the past 1 month, activity restriction status, and stress recognition rate. Considering its clinical unreliability, height was excluded from the feature selection process. Ultimately, a total of 13 features were selected for analysis.

### Model performance and feature importance

**Fig 2:**
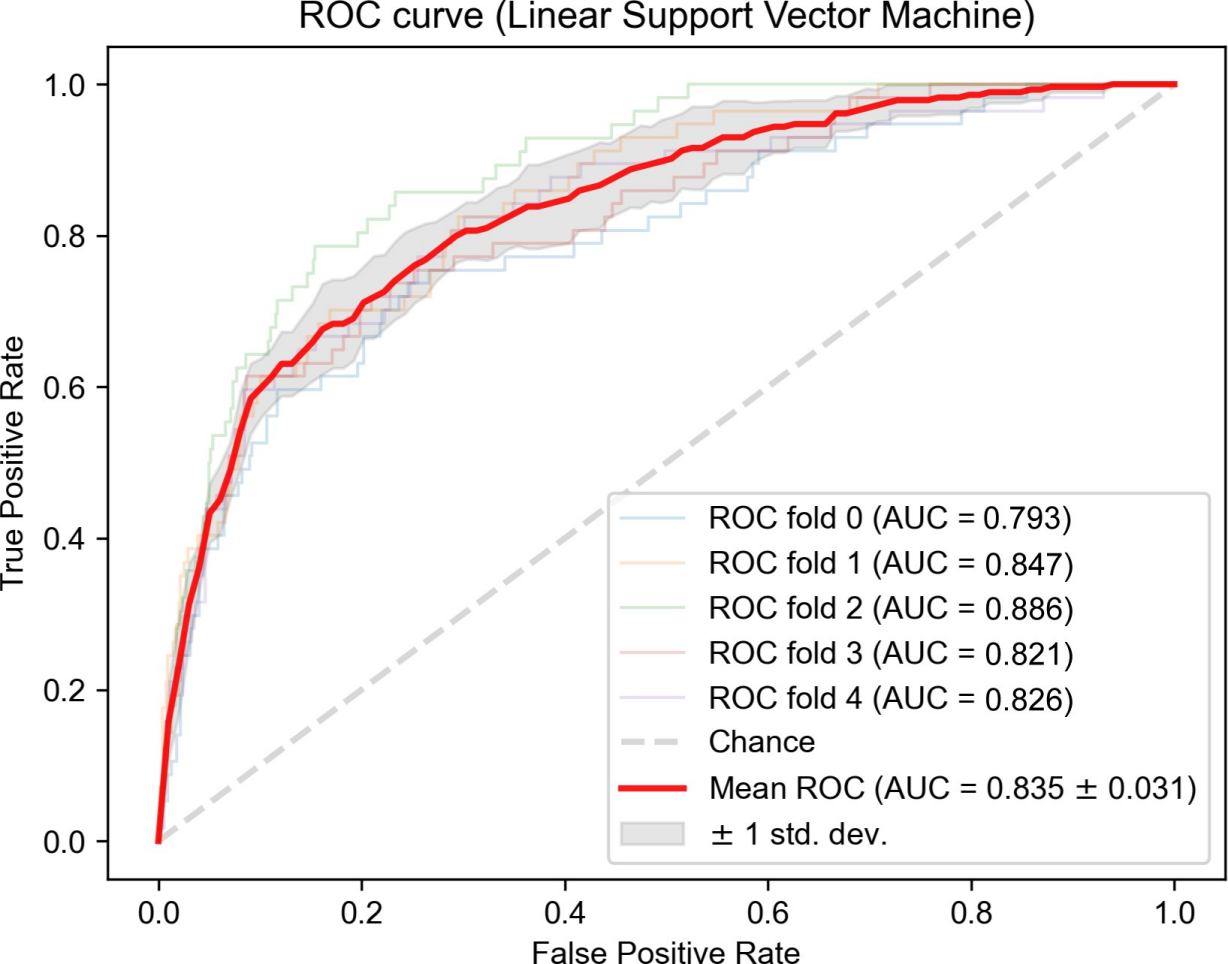
Area under the curve (AUC) of the receiver operating characteristic curve with five-fold cross-validation of the best-performing model, linear support vector machine (SVM). The averaged AUC (95% confidence interval) and accuracy of the model with SVM are 0.835 (0.730–0.901) and 0.879, respectively.

The performance of the ML models is presented in Table 2. The linear SVM performed best (AUC = 0.835, 95% CI = 0.730–0.901) in classifying depression in patients with DM among the six algorithms (Fig 2). The RF model had a slightly lower AUC (AUC = 0.819, 95% CI = 0.731–0.891) than the SVM model, and the KNN model had the worst performance (AUC = 0.648, 95% CI = 0.543–0.850). The ensemble models, including LightGBM (AUC = 0.796, 95% CI = 0.718–0.864), XGBoost (AUC = 0.793, 95% CI = 0.748–0.839), and AdaBoost (AUC = 0.804, 95% CI = 0.685–0.879), exhibited similar performance. Based on the model performance, we selected the model with the SVM algorithm to classify depression in patients with DM.

**Table 2:** Classification performance via five-fold cross validation of algorithms.

Algorithms	AUC	(95% CI)	ACC	Precision	Recall	F1 score
RF	0.819	(0.731–0.891)	0.903	0.716	0.628	0.649
KNN	0.648	(0.543–0.850)	0.865	0.595	0.625	0.596
SVM	0.835	(0.730–0.901)	0.879	0.628	0.694	0.651
LightGBM	0.796	(0.718–0.864)	0.898	0.676	0.601	0.623
XGBoost	0.793	(0.748–0.839)	0.904	0.711	0.616	0.644
AdaBoost	0.804	(0.685–0.879)	0.898	0.708	0.633	0.647

AUC, area under the receiver operating characteristic curve; CI, confidence interval; ACC, accuracy; RF, random forest; SVM, linear support vector machine; LightGBM, light gradient boosting model; XGBoost, Extreme Gradient Boosting; AdaBoost, adaptive boosting model.

The permutation feature importance results are presented in Table 3. The results showed that the most important feature was subjective health status, followed by level of general stress awareness; stress recognition rate; average monthly income; triglyceride level (mg/dL); activity restriction status; EuroQoL: usual activity and lying in a sickbed in the past 1 month; EuroQoL: pain / discomfort, self-care, and physical discomfort in the last 2 weeks; and EuroQoL: mobility and chewing problems.

**Table 3:** Permutation feature importance for model with linear support vector machine.

No	Features	Description	Weight
1	D_1_1	Subjective health status	0.043 ± 0.012
2	BP1	Level of general stress awareness	0.023 ± 0.008
3	mh_stress	Stress recognition rate	0.019 ± 0.007
4	ainc	Average monthly income	0.018 ± 0.021
5	HE_TG	Triglyceride (mg/dL)	0.012 ± 0.007
6	LQ4_00	Activity restriction status	0.01 ± 0.007
7	LQ_3EQL	EuroQoL: Usual activity	0.009 ± 0.012
8	LQ1_sb	Lying in a sickbed in the past 1 month	0.007 ± 0.002
9	LQ_4EQL	EuroQoL: Pain / discomfort	0.005 ± 0.004
10	LQ_2EQL	EuroQoL: Self-care	0.001 ± 0.001
11	D_2_1	Physical discomfort in the last 2 weeks	0.000 ± 0.001
12	LQ_1EQL	EuroQoL: Mobility	0.000 ± 0.001
13	BM7	Chewing problem	-0.005 ± 0.006

## Discussion

This study aimed to create ML models that can identify depression in patients with DM and to determine the best-performing model by assessing multiple ML algorithms while investigating features related to depression. The results showed that the SVM model had the best performance (cross-validated AUC, 0.835). The following 13 features were identified as important for classifying depression in patients with DM: subjective health status, followed by level of general stress awareness; stress recognition rate; average monthly income; triglyceride level (mg/dL); activity restriction status; EuroQoL: usual activity and lying in a sickbed in the past 1 month; EuroQoL: pain / discomfort, self-care, and physical discomfort in the last 2 weeks; and EuroQoL: mobility and chewing problems.

Permutation feature importance revealed that subjective health status was the most significant features of the model. A previous study demonstrated that patients with diabetes had lower self-perceived health, psychological well-being, and quality of life compared with those without diabetes. Factors that contribute to this include being female, having depression, not exercising, and being obese [25]. Another study indicated a weak relationship between HbA1c level and quality of life. However, symptoms of depression in T2DM are associated with a significantly worse health status and quality of life [26]. Thus, the importance of caring for both DM and depression lies in improving individuals’ quality of life and perception of their health status. The current findings, which include a large sample size and the use of ML algorithms, support the findings of previous studies.

The present ML model suggests that stress awareness and average monthly income are important features for classifying depression in individuals with diabetes. Being diagnosed with diabetes, planning suicide for a year, receiving counseling for mental problems for a year, and being aware of stress significantly impacted the level of depression [27]. The risk of DM is significantly higher in lower-income groups than in higher-income groups [28]. Additionally, lower personal income was associated with major depression and depressive symptoms among adults with DM [29]. A Danish nationwide study revealed the notable prevalence of emotional problems among adults with early-onset T2DM [30]. The primary cause of those problems was attributed to perceived stress or depressive symptoms linked to socio-economic factors such as unemployment, low education level, and living alone. The ML model confirms that stress awareness and average monthly income are important features for classifying depression in individuals with diabetes. Therefore, it is crucial to emphasize specialized care for stress management, considering the social status of individuals, to address both diabetes and depressive symptoms effectively.

Another important feature to classify depression in patients with DM is activity restrictions, including quality of life in usual activity, lying in a sickbed in the past 1 month, and activity restriction status. DM and depression generally have a negative effect on patient-initiated activities, such as less physical activity, unhealthy diet, and lower adherence to oral medications [31]. According to a systematic review and dose–response meta-analysis of prospective cohort studies, higher levels of physical activity were associated with a substantially lower incidence of T2DM in the general population [32]. We found that patients with diabetic complications were less likely to engage in physical activity and had poorer self-rated health compared with those without complications. In particular, the presence of DM complications, such as cardiovascular disease, neuropathy, foot ulceration, retinopathy, and nephropathy, can make it challenging to meet the recommended exercise [33]. Consequently, patients with DM fail to engage in appropriate self-care behaviors, including proper physical activity, because of their symptoms and complications, and less physical activity may increase the risk of depression. However, our results do not support a causal relationship between DM and depression. Further longitudinal studies are required to understand the mechanisms and causal directions of activity restriction and depression. However, when developing treatment plans for depression in patients with DM, it is essential to address both the underlying causes of activity restriction and the resulting effects on mental health.

Although comparing the performance of our models may be challenging owing to dataset and analysis variations, classifying depression among patients with DM showed improved performance. The SVM algorithm had an AUC of 83.5% and an ACC of 87.9%. In a previous ML study, the AUC of logistic regression model was 81% [8], and another study showed that the model using SVM had an ACC of 96.875% to classify depression among patients with T2DM [9]. Furthermore, our model’s strengths lie in its potential for expansion. The KNHANES is conducted annually; therefore, our model can incorporate additional features or new data for future research or practical applications. Additionally, Boruta, an algorithm-based technique, was employed for feature selection in our ML model. It can be beneficial for recommending approaches for developing ML models to diagnose other complications associated with DM.

The present dataset was cross-sectional, and there are limitations in understanding the progressive pathways of depression. According to Park, Katon [34], patients with DM who are depressed have a considerably higher risk of death, and early identification and treatment of depression may enhance health outcomes. Further studies using longitudinal data are necessary to develop a model for detecting and predicting the early stages of depression in patients with DM. Nonetheless, the results of the present study, based on various features and a larger sample size, may serve as an initial step for the development of an ML model for predicting depression at an early stage among patients with DM.

The present study has some limitations. First, the KNHANES is a cross-sectional study; therefore, we could not measure the prognosis of the disease or future occurrence of depression in the sample, and the description of the causal effect is limited. Second, we acknowledge the absence of an external validation set in the present study. However, KNHANES is a cross-sectional national survey conducted annually. Therefore, in future research, it would be beneficial to utilize upcoming datasets from KNHANES for validating our model. Third, the definitions of DM and depression relied on self-reporting. Forth, patients with T1DM could be included in this study. The KNHANES does not have a question about DM type; we eliminated patients with DM who were diagnosed before the age of 30 years. Further studies are required to determine the relationship between DM and depression in patients with T1DM.

Despite these limitations, the present study aimed to develop ML models that diagnose depression in patients with DM based on the KNHANES, to determine the best performing model by evaluating multiple ML algorithms, and to investigate features related to depression.

## Conclusion

The SVM model shows the best performance in classifying depression in patients with DM. In addition, subjective health status, followed by level of general stress awareness; stress recognition rate; average monthly income; triglyceride level (mg/dL); activity restriction status; EuroQoL: usual activity and lying in a sickbed in the past 1 month; EuroQoL: pain / discomfort, self-care, and physical discomfort in the last 2 weeks; and EuroQoL: mobility and chewing problems were identified as significant features for effectively classifying depression in this population. The present findings may provide clinicians with a better understanding of the relationship between DM and depression using ML techniques and provide an initiative to develop a further predictive model for the early diagnosis of depressive symptoms in patients with DM.

## Supporting information

S1 File Model hyperparameter table. (DOCX)
