# Supplementary material for: Machine learning‐based identification and related features of depression in patients with diabetes mellitus based on the Korea National Health and Nutrition Examination Survey: A cross-sectional study
Source: PLoS One. 2023 Jul 13;18(7):e0288648. doi: 10.1371/journal.pone.0288648 (PMC10343154; doi:10.1371/journal.pone.0288648)
Supplement: S1 File — (DOCX) [file pone.0288648.s001.docx]

| RF | ‘n_estimators’: [300, 400, 500, 600]  ‘max_depth’: [12, 14, 16, 18]  ‘min_samples_leaf’: [2, 4]  ‘min_samples_split’: [2, 4, 6] |
| --- | --- |
| KNN | ‘n_neighbors’: list(range(1, 20))  ‘weights’: [‘uniform’, ‘distance’]  ‘metric’: [‘euclidean’, ‘manhattan’, ‘minkowski’] |
| SVM | ‘C’: [0.01, 0.1, 1.0, 10.0, 100.0] |
| LightGBM | ‘num_leaves’: [20, 40, 60, 80, 100]  ‘min_child_samples’: [5,10,15]  ‘max_depth’: [-1, 5, 10, 20]  ‘learning_rate’: [0.05, 0.1, 0.2]  ‘reg_alpha’: [0, 0.01, 0.03] |
| Xgboost | ‘n_estimators’: [100, 200, 300, 400, 500]  ‘learning_rate’: [0.01, 0.05, 0.1, 0.15] |
| Adaboost | ‘n_estimators’: [10, 50, 100, 500]  ‘learning_rate’: [0.0001, 0.001, 0.01, 0.1, 1.0] |

**Table S1. Model hyperparameters**
